# Heritability estimation of conventional cardiovascular disease risk factors in Asian Indian families: The Calcutta family study

**DOI:** 10.4103/0971-6866.64944

**Published:** 2010

**Authors:** Arnab Ghosh, Rupak Dutta, Angshuman Sarkar

**Affiliations:** Biomedical Research Laboratory, Department of Anthropology, Visva Bharati University, Santiniketan, West Bengal, India; 1 Departments of Anthropology and Statistics, Visva Bharati University, Santiniketan, West Bengal, India

**Keywords:** Obesity, cardiovascular disease, family study, heritability, Asian Indians

## Abstract

The genetic causes of the components of cardiovascular disease (CVD) risk factors and their intercorrelation are indeed complex and only partly understood. Keeping this view in mind, the present work was undertaken to estimate the heritability of conventional CVD risk factors using family study method. A total of twenty-four nuclear families inhabiting in Calcutta and adjacent areas was chosen randomly. Up to first degree relatives including father, mother and other sibs of the *proband* were considered as participants in the study. Anthropometric measures namely height, weight, waist circumference as well as skinfold thickness at biceps, triceps, subscapular and suprailiac were obtained using standard techniques. Body mass index (BMI), percentage of body fat (PBF), fat mass (FM), waist-hip ratio (WHR), sum of four skinfolds (SF_4_ ), arm muscle circumference (AMC), arm muscle area (AMA), arm fat area (AFA), systolic (SBP) and diastolic blood pressure (DBP) were also considered. To estimate’heritability’ in the study, the mid parent-offspring model was used where’heritability’ (h^2^ ) was equivalent to regression co-efficient (b). The regression sum of square (RSS) and total sum of square (TSS) ratio was also calculated both for mid parent-offspring and single parent-offspring. This ratio was considered as a measure of’heritability’ in the study with consideration that RSS is the variation due to genetic factor and the TSS is due to genetic and other additive factor. It was observed that the estimated heritability for BMI ranges from 0.69 to 0.31 using mid-parent off spring model while the range using single parent-offspring model was from 0.40 to 0.16. The range of heritability for SBP in mid parent-offspring model was 0.16 to 0.44 and 0.05 to 0.54 for single parent-offspring model. To conclude, it seems reasonable to argue that in the study a moderate to high h^2^ was evident for body fat level, body composition and blood pressure measures which indicate a moderate to high aggregation of gene(s) in the family.

## Introduction

A commonly used measure to quantify the extent to which the familial aggregation of diseases due to genetic factors is the’heritability’. In human studies, heritability has the intuitive appeal of quantifying genetic effects without necessarily knowing the mode of inheritance of a trait.[1] Heritability is frequently estimated for complex diseases or traits such as type 2 diabetes mellitus (T2DM), coronary heart diseases (CHD), and serum lipid levels, which are phenotypes reflecting the interplay of genetic and environmental factors.[2] The frequent co-occurrence of T2DM, obesity, and other cardiovascular diseases (CVD) risk factors (e.g., dyslipidemia, hypertension, and chronic inflammation etc.) has led to the hypothesis that these conditions arise from a common antecedent – a concept formalized as metabolic syndrome.[3,4]

Continuous traits or phenotypes (height, body mass index, serum lipid levels, etc) in a population usually have an approximately normal distribution characterized by a mean and a variance. The total variance can be partitioned mathematically into components: a genetic component and an environmental component. The observed correlation between insulin resistance and obesity could be caused by causal associations, by common environmental or genetic backgrounds (pleiotropy), or both causation and common etiology. However, a causal association apparently cannot explain the entire correlation between obesity and insulin resistance.[5–7] Alternatively, genetic or environmental factors common to both insulin resistance and adiposity indices, such as body mass index (BMI), waist-hip ratio (WHR), fat mass (FM), skinfold measures, and trunk-to-extremity ratios, have been reported.[6,8–10] Results from the quantitative genetic analyses have been well supported by molecular findings that indicate genetic influences on both insulin resistance and BMI.[11,12]

In fact, heritability reflects all possible genetic contributions to populations’ phenotypic variances. Included are effects due to act epistatically (multi-genic interactions) as well as maternal and paternal effects where individuals are directly affected of their parents’ phenotype.[1] There are four major designs for inferring the heritability of a quantitative trait parent–offspring, mid parent-offspring, half sib family, and full-sib family designs.[13] Traditionally, to estimate the heritability for a quantitative trait of interest, measurements are taken directly on parents and offspring. This is followed by regression of offspring measurements on parent measurements; the slope of the regression is proportional to the heritability of the trait.[14]

The genetic causes of the components of metabolic syndrome (MS) and their intercorrelation are complex and still only partly understood. Genetic epidemiological studies.[9,15] have begun the search for genetic variants that predispose Mexican Americans to metabolic diseases. However, a few research works have been undertaken regarding the heritability estimation of metabolic syndrome using family study method. In a family study in Northern Manhattan, USA, the heritability of MS was 24% and ranged from 16 to 60% for its five components.[15] A longitudinal family heart study in USA, the MS had increased noticeably from 19.7 to 42.5% with positive family history for conventional CVD risk factors.[16]

However, in India, studies regarding the estimation of heritability of conventional CVD risk factors are virtually absent. Keeping this view in mind, the present work was undertaken to estimate the heritability of conventional CVD risk factors using family study method.

## Materials and Methods

### Study population

The present study was a cross-sectional one, comprising twenty four (24) nuclear families inhabiting in Calcutta and adjacent areas. The study was a part of’Childhood Obesity and Health Research’ project. The probands were randomly selected from the entire data set of school children and adolescents which were collected in the above said project. Before the actual commitment of the work, the probands were contacted over telephone and the parents of the probands were convinced to carry on this family study. Up to first degree relatives including father, mother and other sibs of the proband were considered as the participants in the study. The study was undertaken from July 2008 to February 2009. The Institutional Ethics Committee (IEC) of the Human Genetic Engineering Research Center (HGERC), Calcutta, India has had approved the study. Written consent was obtained from participants prior to actual commencement of the study.

### Socioeconomic characteristics

The name, age (date of birth), sex, maturation status as well as information on socioeconomic characteristics including family type, gross family income and expenditure, occupation and education etc., were obtained using an open ended schedule.

### Anthropometric and body composition measures

Height, weight, waist circumference as well as skinfold thickness at biceps, triceps, subscapular and suprailiac were obtained using standard techniques.[17] Height and weight of lightly clothed subjects were measured to the nearest 0.1 kg and 0.1 cm, respectively. Skinfolds thicknesses at biceps, triceps, subscapular and suprailiac were measured on the left side of the body to the nearest 0.2 mm using a Holtain skinfold caliper (Holtain Corporation, UK). Sum of four skinfolds (SF_4_ ) was calculated subsequently. Circumference of minimum waist (MWC) and hip (MHC) was measured with an inelastic tape to the nearest 0.1 cm. Waist-hip ratio (WHR) was computed accordingly. Percentage of body fat (% BF) and body mass index (BMI) were measured using an Omron body fat analyzer (Omron Corporation, Tokyo, Japan). It is noteworthy to mention that the Pearson’s correlation coefficient (r) between analyzer operated BMI and manually calculated BMI (weight in kg/height in m^2^) was 0.92 (*r* = 0.92; *P*< 0.0001). The arm muscle circumference (AMC), arm muscle area (AMA) and arm fat area (AFA) were computed using standard equation.

### Blood pressure variables

Left arm blood pressure (BP) was taken from each participant with the help of an Omron M1 digital electronic blood pressure/pulse monitor (Omron Corporation, Tokyo, Japan). Two forenoon BP measurements were taken and averaged for analysis. A five minutes relaxation period between measurements was maintained for all individuals. The working condition of the instrument was checked periodically with the help of a mercury sphygmomanometer and a stethoscope (auscultator procedure). All BP measurements were taken at room temperature. Systolic (SBP) and diastolic blood pressure (DBP) was defined as the points of appearance (Phase I) and disappearance (Phase V) of the korotkoff sounds, respectively.

### Statistical analyses

Descriptive statistics such as mean and standard deviation (SD) were undertaken for all the variables. For estimating heritability in the present study, the mid parent-offspring model has been used where, heritability (h^2^ ) is equivalent to regression co-efficient (b). Therefore, the regression sum of square (RSS) and total sum of square (TSS) ratio has been calculated both for mid parent-offspring and single parent-offspring. This ratio is being considered as a measure of heritability in the study as the RSS is the variation due to genetic factor and the TSS is due to genetic and other additive factor.

All statistical analyses were performed using the SPSS (PC + version 16). A *P* value of <0.05 (two tailed) was considered as significant.

## Results

The mean and standard deviation (SD) of anthropometric, body composition and blood pressure variables are presented in Table 1. The mean age of fathers, mothers, sons and daughters were 48.67(5.67), 40.42(5.87), 14.44(1.59) and 12.55(2.91) years, respectively. The mean mid-parent BMI was 23.45 (SD=2.73).

**Table 1:** Descriptive statistics of anthropometric, body composition and blood pressure measures in the study population.

Variables	Father	Mother	Son	Daughter	Mid-Parent
	mean (SD)	mean (SD)	mean (SD)	mean (SD)	mean (SD)
Age (years)	48.67 (5.67)	40.42 (5.87)	14.44 (1.59)	12.55 (2.91)	44.54 (5.77)
BMI (kg/m^2^)	23.14 (3.02)	23.76 (3.50)	21.21 (3.87)	20.70 (5.06)	23.45 (2.73)
MWC (cm.)	77.48 (7.52)	77.81 (10.46)	69.24 (8.50)	70.95 (12.17)	77.65 (7.87)
SF4 (mm.)	35.89 (11.49)	54.39 (16.99)	43.95(24.92)	62.35 (31.46)	45.14 (13.03)
PBF	24.55 (4.41)	32.29 (3.90)	19.70 (9.27)	26.23 (5.79)	28.42 (3.45)
WHR	0.88 (0.03)	0.82 (0.06)	0.79 (0.05)	0.81 (0.05)	0.85 (0.03)
AMC (cm.)	23.87 (2.04)	21.72 (2.98)	21.34(2.49)	18.51 (2.46)	22.8 (1.69)
AMA (cm^2^)	44.71 (7.72)	38.16 (10.72)	36.62 (8.32)	27.63 (7.24)	41.44 (6.21)
AFA (cm^2^)	11.06 (6.31)	14.45 (5.91)	11.78 (5.29)	12.85 (4.51)	12.76 (5.67)
SBP (mmHg)	129.56 (14.62)	127.79 (16.49)	120.41 (14.11)	112.04 (7.56)	128.68 (13.46)
DBP (mmHg)	85.77 (7.94)	85.06 (12.26)	78.06 (8.59)	77.23 (11.23)	85.45 (8.16)

BMI= Body mass index; MWC= Minimum waist circumference; SF4= Sum of four (biceps + triceps + subscapular + suprailiac) skinfolds;

PBF= Percentage of body fat; WHR= Waist-hip ratio; AMC= Arm muscle circumference; AMA= Arm muscle area; AFA= Arm fat area;

SBP= Systolic blood pressure; DBP= Diastolic blood pressure RSS/TSS= Regression sum of square/ total sum of square SD= Standard deviation

The measures of heritability (h^2^) using mid parent-offspring and single parent-offspring model in the study are represented in Table 2. For mid parent-offspring model, both the regression coefficient (b) and the ratio of regression sum of square (RSS) to total sum of square (TSS) had been computed to estimate h^2^ . Whereas, for single parent-offspring model only the ratios of RSS to TSS had been calculated for all the expected combinations (father-son, father-daughter, mother-son and mother-daughter). It was observed that the estimated heritability for BMI ranges from 0.69 to 0.31using mid-parent off spring model while the range using single parent-offspring model was from 0.40 to 0.16. In case of percentage of body fat (PBF), the ranges for the above two models were 0.94 to 0.19 and 0.29 to 0.003. For SF_4_, the ranges of heritability for the two models were 0.24 to 0.83 and 0.15 to 0.38, respectively. In case of AMA, the range of heritability from 0.12 to 0.62 for mid parent-offspring model but this range varies from 0.02 to 0.43 using single parent-offspring model. The range of heritability for SBP, in mid parent-offspring model was 0.16 to 0.44 and 0.05 to 0.54 for single parent-offspring model.

**Table 2:** Estimation of heritability using mid parent-offspring and single parent-offspring model in the study.

Variables	B	RSS/TSS	RSS/TSS	RSS/TSS	RSS/TSS	RSS/TSS	RSS/TSS
	(Mid parent -Offspring)	(Mid parent -Son)	(Mid parent Daughter)	(Father - Son)	(Father - Daughter)	(Mother-Son	(Mother - Daughter)
BMI (kg/m^2^)	0.69	0.31	0.54	0.40	0.27	0.16	0.29
MWC (cm.)	0.45	0.25	0.22	0.40	0.13	0.08	0.25
SF4 (mm.)	0.83	0.24	0.28	0.21	0.38	0.20	0.15
PBF	0.94	0.14	0.19	0.29	0.08	0.003	0.21
WHR	0.28	0.05	0.04	0.05	0.15	0.03	0.02
AMC (cm)	0.48	0.15	0.42	0.20	0.18	0.02	0.25
AMA (cm^2^)	0.40	0.12	0.62	0.17	0.43	0.02	0.22
AFA (cmm^2^)	0.45	0.33	0.30	0.36	0.29	0.23	0.25
SBP (mmHg)	0.44	0.16	0.39	0.05	0.22	0.16	0.49
DBP (mmHg)	0.50	0.28	0.29	0.27	0.22	0.22	0.23

BMI= body mass index; MWC= minimum waist circumference; SF4= sum of four (biceps + triceps + subscapular + suprailiac) skinfolds; PBF= percentage of body fat; WHR= waist-hip ratio; AMC= arm muscle circumference; AMA= arm muscle area; AFA= arm fat area; SBP= systolic blood pressure; DBP= diastolic blood pressure, B= regression co-efficient, RSS/TSS= Regression sum of square/ total sum of square

## Discussion

The measures of heritability to evaluate the genetic contributions for quantitative traits must be interpreted with great caution. In general, heritability is not a reliable measure to compare the relative importance of genes to explain differences in disease occurrence between different populations or to compare the genetic contribution to different traits.[13]

In a study in Washington D. C., USA, it was observed that the maximal heritability for abdominal visceral fat (AVF), before and after adjustment for total fat mass (FM), was 42 and 50%, respectively while for insulin, it was 21%.[10] Interestingly, 29% of the familial influences on insulin were also common to AVF, whereas 14% of the familial influences on AVF were shared by insulin.[8] Furthermore, after AVF was adjusted for total FM, these common familial influences were increased to 48and 20%. In a family study in Northern Manhattan, USA, the heritability of MS was 24% and ranged from 16% to 60% for its five components.[15] There has been some discussion about which adiposity phenotype is the best correlate of insulin resistance. AVF seems to be a better predictor of insulin resistance and T2DM as compared to other adiposity indices, although several studies have not controlled the AVF data for the concomitant effects of FM[2] hence there are contradictory results. In our study, a moderate to high h^2^ was evident for body fat level, body composition and blood pressure measures which indicate a moderate to high aggregation of gene(s) in the family.

The major limitation of the study was that it was performed on a relatively small family size. The lack of assessment of plasma lipids further limits the study results. Owing to considerable ethnic and cultural heterogeneity in Asian Indian population, similar studies on migrant Asian Indian families would yield valuable information in the *nature-nurture* interaction involved in the conventional CVD risk factors.
